# Individual differences effects on the psychological refractory period

**DOI:** 10.1186/2193-1801-2-368

**Published:** 2013-08-01

**Authors:** Maude Laguë-Beauvais, Christine Gagnon, Nathalie Castonguay, Louis Bherer

**Affiliations:** Université du Québec à Montréal : Département de Psychologie, Université du Québec à Montréal (UQÀM), C.P. 8888, succursale Centre-ville, Montréal, Québec, H3C 3P8 Canada; Centre de Recherche de l’Institut Universitaire de Gériatrie de Montréal, 4545 Chemin Queen-Mary, Montréal, Québec, H3W 1W4 Canada; PERFORM Centre, Concordia University, 7141 Sherbrooke St. W, Montreal, Quebec, H4B 1R6 Canada

**Keywords:** Psychological refractory period, Divided attention, Gender, Processing speed, Neuropsychological predictors, Individual differences

## Abstract

The goal of this study was to assess the impact of individual neuropsychological differences on the ability to share attention between concurrent tasks. Participants (n = 20) were trained on six single task practice sessions and dual-task was assessed with reaction time performance on a psychological refractory period (PRP) paradigm. Neuropsychological test scores were also acquired. Furthermore, one of the known variables that can influence performances on neuropsychological tests is gender, which was added as a potential predictor. Results show that the small PRP group was associated with better performances in processing speed, inhibition, flexibility and working memory on neuropsychological tests. Gender also had an impact on the PRP, males having a lower PRP than females. A multiple regression was performed to determine which variables explained the most PRP duration, which showed that 49.1% of the variance of the PRP length could be explained by gender, reaction times of the PRP practice trials at the sixth session, the denomination and flexibility conditions of the Modified Stroop Task as well as results on the Symbol Search Test. Gender was the variable that explained the PRP variance the most (23%). Processing speed also seemed to be a great determinant of the PRP as well as the ability to alternate between task-sets as assessed by the Flexibility condition of the Modified Stroop Task. Thus, this study reveals that good performances on certain neuropsychological tests could predict one’s ease to manage two tasks simultaneously with a higher chance for males to perform better.

## Background

As society emphasizes productivity more and more, the ability to perform two things at the same time has become a major concern for both public and scientific interest. This is not a trivial question if we consider that the ability to carry out two tasks at once might sometimes have deadly consequences (e.g., driving while talking to cell phone). While some researchers argue that perfect-time sharing between two tasks is possible (Schumacher et al. 2001), others claim that the fundamental structure of human cognition limits multi-tasking (Pashler 1993; Pashler and Johnston 1998; Tombu and Jolicoeur 2004; Ruthruff et al. 2003; Hartley and Maquestiaux 2007) Surprisingly, individual differences in neuropsychological functions are one major source of explanation for discrepancy among studies that has never been systematically investigated. The goal of the present study was to investigate whether individual differences in attentional control come into play in individuals’ ability to share their attention between concurrent tasks.

Divided attention is defined as the capacity of sharing attention between two or more incoming stimuli and it has been associated with Baddeley’s central executive (Pashler 1993). According to Baddeley ( 1996), one of the roles of the central executive system is to act as an attentional controller. This controller can focus on specific information, while rejecting information from other sources that are not task-relevant and it is greatly involved in divided attention tasks. One’s capacity to efficiently control attention has further impact on related systems of executive functioning, such as working memory (Cowan et al. 2005; McDowell et al. 1997). Thus, it is important to know the limits of this system to further our knowledge in this area, and studies of divided attention are often used to do so (Coull 1998). Furthermore, many dual-task paradigms are complex and involve a variety of perceptual, memory and motor processes, and do not allow localizing the source of the dual-task effect. The effect depends on the ability to sustain inhibition between upcoming stimuli, to synchronize concurrent output or switch rapidly between tasks, using buffer-switch strategies (Monsell 2003). For these reasons, some authors favour the Psychological Refractory Period (PRP) paradigm. In the PRP paradigm, participants perform two reaction time (RT) tasks (e.g., identifying a letter and discriminating between a high or low tone) in sequential order. The delay between the two RT tasks varies, allowing a measurement of the source of the interference between tasks, such as the modality of stimulus presentation, the cognitive processes employed during task performance, and/or the response processes. This delay also creates a bottleneck where the first answer must be processed before another one is (Pashler 1993; Meyer and Kieras 1997a, b). A common finding in PRP studies is that reducing the delay between the tasks impairs performance in the second task. This has often been used as an argument that perfect time sharing between two tasks is not possible and that at some point in time, execution of the second task must await the completion of the first task. Interestingly, recent studies have reported individual differences in the ability to divide attention between the two tasks in a PRP paradigm (Maquestiaux et al. 2004; Maquestiaux et al. 2008; Ruthruff et al. 2006). For example, Ruthruff et al. ( 2006) found that, after extensive training on the second task, some persons are capable of perfect time sharing between tasks. For those participants, the execution of the second task does not suffer from reducing the delay between the two tasks. Ruthruff et al. ( 2006) suggested that these participants have the ability to bypass the central bottleneck of information processing, a common explanation for the classical PRP effect. These results are consistent with previous findings. In fact, using a somewhat different task in which the two RT tasks must be performed at the same time instead of following a sequential order, Schumacher et al. ( 2001) found that some individuals achieved perfect time sharing after five practice sessions. Perfect time sharers could execute a task in dual-task condition as fast as they would when the task was performed alone. Thus, evidences reported so far suggest that extensive practice can improve the ability to perform two independent tasks at the same time, but that strong individual differences exist in the ability to share attention between tasks even after substantial practice.

Evidences for individual differences in dual-tasks suggest that these might be related to differential abilities in executive functions. Behavioural studies that explored the relation between dual-tasks performance and neuropsychological tests support the idea that individual differences in executive functions can predict the ability of an individual to perfectly share attention between tasks. However, it seems that the link between neuropsychological testing of executive functioning and dual-task is not as straightforward as one might wish. On the one hand, some studies found that the link between the two is rather weak. For example, Bull and Scerif ( 2001) found that the children’s performances on a dual-task based on Baddeley et al. ( 1997) did not correlate with the neuropsychological tests of executive functioning used in the study, except with a facilitation score of the Stroop, which was a subtraction between the congruent condition and the baseline. Results found by McDowell et al. ( 1997), showed that dual-task measures, i.e., press a button when a dot appeared on the screen while doing a digit span task, only correlated to executive functioning tests for patients with traumatic brain injuries, but not in healthy controls. In a factor analysis study conducted on a college population, Miyake et al. ( 2000) found three latent variables (i.e., shifting between mental sets, updating and monitoring of working memory representations and inhibition of dominant responses) that were derived to predict performance on certain tests of executive functioning, including the Operation Span Task and a dual-task. Only the updating variable contributed to the Operation Span task and none of them contributed to the dual-task. On the other hand, other studies have found a relation between neuropsychological testing and dual-task performance. For example, Tun et al. ( 2002) used another type of divided attention paradigm where younger and older adults listened to a target speech in an environment with a competing speech in the background. They found that participants’ performances at the Trail Making Test B, a measure of executive functioning, were correlated with recall accuracy when there were meaningful distractors, but not when the information was presented in a quiet setting without interference. Furthermore, in a study using the Delayed Visual Recognition Task, which is a dual-task using a visual and a verbal task, Holtzer et al. ( 2005) performed a regression analysis to know which neuropsychological factor best predicted dual-task performances in younger and older adults. It seems that dual-task performance was best predicted by the “attention/executive” factor (22.6% of the variance), which included tests such as the Forward and Backward Digit Span, Trail Making Tests A and the subtraction of the Trail Making Test B minus A, and the copy of the Rey Complex Figure.

It thus seems that the relationship between neuropsychological test performances and the ability to divide attention between tasks is equivocal. Studies are scarce and provide inconsistent findings. This could be partly due to the wide variety of dual-task paradigms and methodology used, in which individual differences in attentional control strategy and task prioritization, that might also differ among participants, are never controlled. The PRP paradigm has often been used in an attempt to better control tasks conditions and individual strategy when studying dual-tasking abilities. The aim of the present study was to assess whether performances on neuropsychological tests, particularly those tapping executive functioning, are related to individual differences in perfect time sharing following an extensive training with a PRP paradigm. To accomplish this, we used a modified training procedure of the PRP paradigm inspired by Ruthruff et al. ( 2006) (experiment 2), which showed that perfect time sharing was possible (see Maquestiaux et al., 2008 for complete results of the study), and also administered specific neuropsychological tests. Although the link between divided attention and executive functioning is controversial, we hypothesized that tests measuring attentional inhibition and flexibility will be the best predictors of PRP. Furthermore, one of the known variables that can influence performances on neuropsychological tests is gender. For example, some studies have shown gender differences in attention paradigms (Voyer et al. 1995; Goddard et al. 1998; Hancock et al. 2003) and men are revealed to be faster in the latter. Hence, gender was added as a potential predictor of PRP.

## Results

### PRP paradigm results

**Figure 1:**
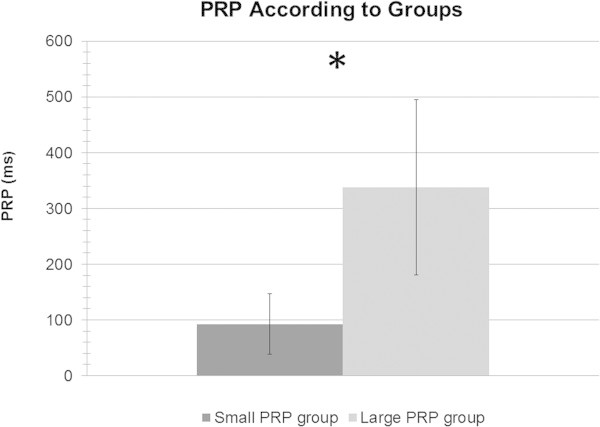
**Time in ms of the large PRP group and the small PRP group.**

The PRP was calculated by subtracting the mean amount of time to answer task 2 for the long stimulus onset asynchrony (SOA ; i.e., 1000 ms) to the mean response time to task 2 for the short SOA (i.e., 15 ms) in the last session. As reported in Maquestiaux et al. ( 2008), there was a main effect of SOA in the last two sessions for task 1, with the 1000 ms SOA response time being longer than the 250 ms SOA response time by 29 ms only. No other SOA comparisons revealed differences for task 1. The PRP effect in task 2 was not compared statistically, but the task 2 response time at the 15 ms SOA was *X* = 588 ms, *SD* = 214 ms, while at the 1000 ms SOA, the task 2 response time was of *X* = 372 ms, *SD* = 93 ms. Thus, we can safely conclude that it was statistically different. A median-split was then performed on PRP values, which created two groups, i.e., a small PRP group (*X* = 92.50 ms, *SD* = 54.03, 7 men and 3 women) and a large PRP group (*X* = 337.90 ms, *SD* = 156.89, 3 men and 7 women; see Figure 1). A one-way ANOVA reveals that on the sixth session of training the small PRP group, *X* = 288 ms*, SD* = 55 ms, was not significantly faster than the large PRP group, *X* = 322 ms*, SD* = 56 ms , *F(1, 19)=*1,88, *p*>.5, meaning that the PRP effect observed is not due to overall response speed to the specific task. According to Ruthruff et al. ( 2006), a simultaneous execution of central stages of attention, i.e., perfect time sharing, is defined by a low PRP (less than 150 ms), a high ratio of inverse response order, and Task 1 reaction times that stay constant no matter what the SOA is. Following these criteria, ten out of the 20 participants could be considered perfect time sharers, corresponding to our participants in our small PRP group. Moreover, a significant difference between our PRP groups was found, the small PRP group having a less pronounced PRP than our large PRP group, *F* (1,19) = 21.87, *p* < .001. Using the task 2 RTs (RT2), a 6 X 2 mixed design repeated ANOVA with SOAs (15, 65, 150, 250, 550 and 1000 ms) and PRP groups (small and large) as factors did reveal a significant interaction between the two factors, F(5, 90) = 15.38, p <.001, and a between group effect between the small and large PRP groups, F(1, 18) = 11.70, p .01. The effects were all found in the large PRP group. In post-hoc comparisons, there were no significant differences in the small PRP group in the RT2 SOAs. According to the literature, we can safely say that participants of the small PRP group have bypassed the central bottleneck after training. One-way analyses of variance (ANOVAs) did not reveal any age, *F* (1,19) = .27, *p* = .66) or education level (*F* (1,19) = .08, *p* = .78) differences between our groups. Consequently, we decided to compare our different groups according to their results on neuropsychological tests.

### Group differences on neuropsychological tests

**Figure 2:**
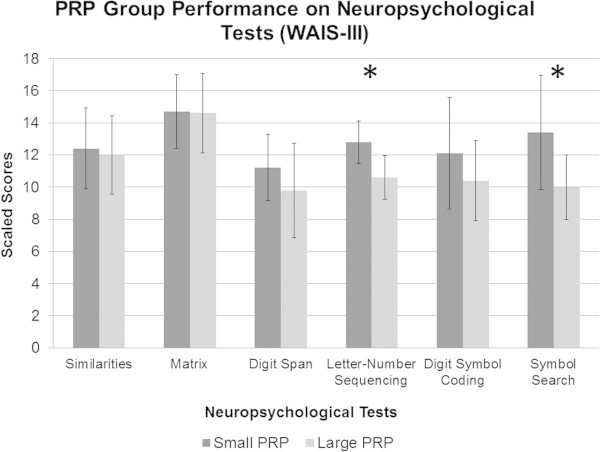
**Scaled scores of neuropsychological subtests of the WAIS-III for the small and large PRP group.**

**Figure 3:**
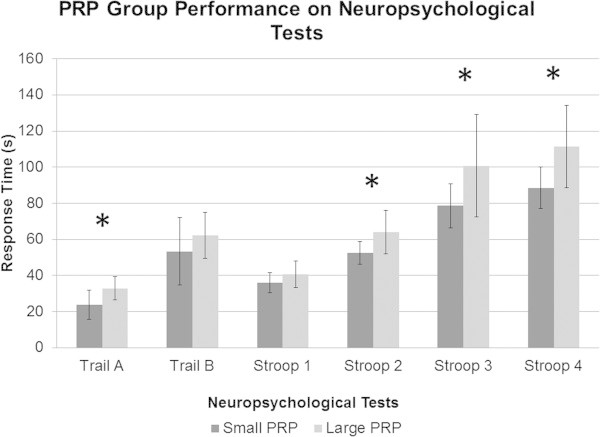
**Response time (s) of neuropsychological tests for the small and large PRP group.**

Results are shown in Figures 2 and 3. Groups did not differ significantly on the lexical access measure (Verbal Fluency Test), *F* (1,19) = .17, *p* = .69, on abstraction measures, i.e., Matrix Reasoning Test, *F* (1,19) = .009 , *p* = .93 and Similarities, *F* (1,19) = .13, *p* = .72, or on attention span with the Digit Span Forward Test, *F* (1,19) = 1.55, *p* = .23, or Digit Span Backward, *F* (1,19) = .14, *p* = .71. However, participants in the small PRP group performed significantly better on various processing speed tests, namely Symbol Search, *F* (1,19) = 7.01, *p* < .05, Trail Making Test A, *F* (1,19) = 7.76, *p* < .05, and on the Denomination condition of the Modified Stroop Test, *F* (1,19) = 7.07, *p* < .05. No significant differences were found on the Digit Symbol Coding Test, *F* (1,19) = 1.57, *p* = .23, and on the Reading condition of the Modified Stroop Test, *F* (1,19) = 2.67, *p* = .12. The small PRP group also performed significantly better on measures of inhibition and flexibility, respectively on the third condition of the Modified Stroop Task, *F* (1,19) = 5.15, *p* < .05, and on the fourth condition as well, *F* (1,19) = 8.05, *p* < .05. Finally, a significant difference was found on the working memory task, i.e., Letter-Number Sequencing, *F* (1,19) = 13.61, *p* < .01.

### Gender differences

**Figure 4:**
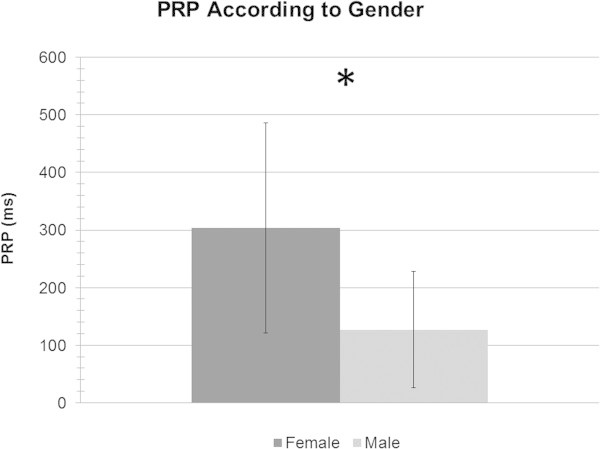
**Time in ms of females and males PRPs.**

To compare PRP values between genders (see Figure 4), we first did an independent sample t-test, which revealed that women were significantly slower than men, *t*(1,19) = 2.69, *p* < .05.

When comparing the effect of gender on neuropsychological tests, females (*X* = 32.6 s, *SD* = 8.24 s) were only slower than males (*X* = 24.1 s, *SD* = 6.69 s) on the Trail Making Test Part A, *F* (1,19) = 6.42, *p* < .05, which is a visual search and processing speed task. All other comparisons between tests were not significant.

### Multiple regression

The aforementioned ANOVAs suggest that cognitive abilities associated with certain neuropsychological tests are necessary to perform well in a PRP paradigm. Thus, to validate this hypothesis and to determine which variable contributed the most to explain PRP, we completed a backward multiple linear regression. The dependent variable was the PRP values whereas the independent variables were the test scores and responses times in which there was a significant difference between small PRP and the large PRP groups, i.e. Letter-Number Sequencing test (working memory), Symbol Search Test (processing speed), Trail Making Test A (processing speed), Modified Stroop conditions, # 2 (processing speed) , # 3 (inhibition), # 4 (flexibility). Furthermore, we decided to add Task 2 RTs of the first and sixth practice sessions as independent variables to know if it was only due to processing speed. A 2 × 2 mixed-subject design ANOVA comparing Task 2 RTs of the first and sixth session between the small and large PRP groups revealed a main effect of practice on Task 2, *F* (1, 18) = 144.54, *p* < 0.001, the participants being faster on the sixth session (*X* = 304.86 ms, *SD* = 56.72 ms) than on the first one (*X* = 469.38 ms, *SD* = 80.60 ms), but no main effect of PRP group (*F* (1, 18) = 1.25, *p* =.28) or interaction (*F* (1, 18) = .04, *p* =.85) between the small and large PRP groups were found, meaning that both groups benefited equally from the practice sessions. Finally, since a significant difference was found on PRP values according to gender, we decided to include this variable in the multiple regression. The best model explained 49.1% of the PRP values, adjusted *R*^*2*^ = 0.491, *F* (4, 19) = 4.67, *p* < 0.01, and included the Task 2 RTs at the sixth session, gender, the Denomination condition and Flexibility condition of the Modified Stroop Test, and results on the Symbol Search Test as well. Squared semi-partial correlations indicated that gender explained 23.0% of the variance associated with PRP values and that the Stroop Denomination condition explained 12.7% of the variance. As for the Stroop Flexibility condition, Task 2 RTs at the sixth session and Symbol Search results explained respectively, 5.1%, 4.0% and 2.6% of the PRP variance. As for the directionality of the relationship between the different independent variables and PRP values, significant standardised beta coefficients revealed that higher RTs on the Stroop Denomination condition are linked to higher PRP values (ß = .72, *t* = 2.18, *p* < .05) and that being a female is also associated with a higher PRP (ß = −.54, *t* = −2.94, *p* < .05), since we had a dichotomic variable score and the females were given a score of 1 and males, a score of 2.

## Discussion

The goal of this study was to identify the impact of individual differences and gender on the psychological refractory period. Neuropsychological tests were used to determine which cognitive functions were associated with the length of the PRP. Moreover, we explored how gender differences and performances on specific cognitive tests could explain the length of the PRP.

We first observed that participants in the Small PRP group performed better on tests involving processing speed, such as the Symbol Search subtest, the Trail Making Test Part A and the denomination condition of the Modified Stroop Test. They also performed better on tests involving inhibition, flexibility and working memory, i.e., the Inhibition and Flexibility condition of the Modified Stroop Task and on the Letter-Number Sequencing subtest. Thus, it would seem that to be able to lower the impact of managing two tasks at once, one must not only be fast, but also have a certain advantage in specific executive and attentional functions. A good hold of task-set in working memory, as well as being able to inhibit automatic responses to a task and easily switch between two tasks seem to help doing two things at the same time effectively. Results are concordant with Tun et al. ( 2002), which found that performances on the Trail Making Test A and B were correlated with recall accuracy in an interference condition, and also with Holtzer et al. ( 2005), which found that performances on a dual-task were related to scores of an attention/executive factor, composed of different measures of the aforementioned functions. We also showed that gender had an impact on the PRP, males having a lower PRP than females. This result is consistent with the literature (Briem and Hedman 1995; Voyer et al. 1995; Goddard et al. 1998; Vecchi and Girelli 1998; Hancock et al. 2003; Saucier et al. 2003), since women are generally more affected by an interfering condition in diverse tasks, which suggests that this could be due to core gender differences in divided attention.

Apart from individual differences in executive functioning, gender differences also appear to have an impact on the ability to divide attention between tasks. For example, Vecchi and Girelli ( 1998; experiment 2) measured the impact of gender on performances on a visuo-spatial task executed concurrently either with a passive interference task (i.e. remembering common words) or with an active interference task (i.e., forming non-words from different syllables). Males were significantly better than females in their performance on the visual task when doing the active interference task. Thus, gender differences in visuo-spatial tasks are found when both tasks must be actively processed. Similar results were found by Saucier et al. ( 2003) in a study where participants realized a visuo-spatial task while either performing an articulatory interference task (repeating the days of the week) or while executing a spatial interference task (tapping pattern with left hand). While males were not affected differently by the interference tasks, women performed less well when doing the articulatory interference tasks. The authors explained this difference with the hypothesis that the visuo-spatial sketchpad would be taxed more quickly in women than in men. Furthermore, gender differences in visuo-spatial ability are well documented, favouring men (Voyer et al. 1995). In a dual-task paradigm in which participants had to respond simultaneously to a social problem solving task and a four-choice reaction time task, two conditions in the reaction time task were used to evaluate the cognitive load (Goddard et al. 1998). The difficulty level (easy and difficult) was manipulated by reducing the delay between the stimuli, changing the response keys during the task and adding feedback on omissions and errors. Results indicated that only females’ performance were affected by the high cognitive load condition, while males’ performance remained unchanged. Results from studies looking at distraction effects of using a phone while doing a crucial driving manoeuvre are less clear. On the one hand, a study from Hancock et al. ( 2003) did not find any differences between genders in younger adults, but older women were slower than their younger counterparts when it came to doing two tasks at the same time. On the other hand, another study found that women’s performances were more affected than men’s performances by cell phone use while driving in a traffic situation in which obstacles had to be avoided (Woo and Lin 2001). Similarly, females’ performances seemed to be affected by material manipulation while driving, while no such effect in males’ performances has been observed (Briem and Hedman 1995). Thus, these studies showed that in various divided attention situations, women’s performances seem to be more affected than men’s performances by dual task interference. In the light of these results, it can be suggested that this gender difference is in fact due to a difference in the basic ability to share attention between two tasks.

Finally, to determine which variables explained the most PRP duration, we performed a multiple regression, which showed that 49.1% of the variance of the PRP length could be explained by gender, Task 2 RTs at the sixth session, the denomination and flexibility condition of the Modified Stroop Task as well as results on the Symbol Search Test. Surprisingly, gender was the variable that explained the PRP variance the most (23%). Processing speed also seemed to be a great determinant of the PRP as well as the ability to alternate between task-sets as assessed by the Flexibility condition of the Modified Stroop Task. Contrary to Holtzer et al. ( 2005), the only executive function measure that seems to predict PRP performance is the flexibility condition of the Modified Stroop task. These diverging results may be attributable to the fact that the dual-task employed in their study had a strong working memory component and that older adults as well as younger adults were included in the principal component analysis.

One aspect that was not explored in this study, but that is known to have an impact on cognitive demands is personality (Berbner 1998; Kumari et al. 2004). Berbner ( 1998) found that extroverts were faster on both tasks in a PRP paradigm and that the PRP was also shorter for extraverts than introverts. Kumari et al. ( 2004), did not find any relationship between personality traits and cognitive demands using an *n*-back task, but did show that the higher the extravert rating was the grater the activation was in regions associated with attention, online monitoring, error detection and response execution. Further studies should include measures of personality in order to dissociate the impact of gender, executive functioning, processing speed, and personality on one’s ability to effectively share attention between two tasks.

The main limitation of this study comes from the fact that neuropsychological tests were given randomly throughout the entire training program, which might have influenced the performances on the different tests. On the other hand, it has been shown that training performances on a specific task will only transfer on the performances involving the same cognitive function (Ball et al. 2007) in this case, processing speed. Moreover, the number of participants included in the multiple regression is low, which can increase the amount of type II errors. The same error may occur in the computed ANOVAs, since the groups only contain ten participants each.

## Conclusion

In sum, this study reveals that good performance on specific neuropsychological tests could predict one’s ease to manage two tasks simultaneously with a higher chance for males to perform better. Good performance in tests assessing speed of processing and flexibility are determinant in the ability of sharing attention between two concurrent tasks. These findings can have significant impacts when designing adapted intervention programs to improve attentional control and divided-attention in older adults. Further research with more participants is needed to confirm the obtained results. Maintaining and enhancing these functions as we grow older is crucial as they play a critical role in mobility, risk of falls, and instrumental activity of daily living in these populations ( Fraser and Bherer in press).

## Method

### Participants

Twenty younger adults (*M* = 24.6 years, *SD* = 2.5 years, range = 20–31 years) were recruited from the *Université du Québec à Montréal* and the *Institut universitaire de gériatrie de Montréal* to participate in this study. The sample was composed of 10 men and 10 women, generally highly educated (*M* = 17.6 years, *SD* = 1.6 years). All participants reported good health (*M* = 4.6, *SD* = 0.5) on a 5-point Likert scale, 1 being “bad” and 5, “very good”. According to their own self-report, four of the participants were left-handed (3 men and 1 woman) and 16 (7 men and 9 women) of them were right-handed. Participants were screened for normal or corrected-to-normal vision and hearing using self-report. None of them reported any difficulties in discriminating the auditory and visual stimuli presented in the experiment. They also had no history of neurological diseases and did not take any medication known to affect cognition. They were paid 10$ CAD per hour for their participation. This study was approved by the ethics committee of the *Centre de recherche de l’Institut universitaire de gériatrie de Montréal*.

### Procedure and material

Each of the 20 participants performed nine sessions that lasted about an hour each, at a rate of one per day maximum in a span of three weeks. Throughout the sessions, the different neuropsychological tests were administered to the participants. The first training phase consisted of six extensive auditory Task training sessions. The PRP paradigm second training phase consisted of three dual-task sessions.

#### Neuropsychological testing

The neuropsychological assessment consisted of a history questionnaire with questions on health and demographics, as well as nine tests, evaluating lexical access, capacity of abstraction, attentional span, processing speed, inhibition, flexibility, and working memory (see Table 1).

**Table 1:** **Cognitive functions that were covered during testing and the neuropsychological tests associated with each of them**

Cognitive functions	Neuropsychological tests
Lexical access	Verbal fluency
Abstraction	Similarities
	Matrix Reasoning
Attention span	Digit Span
Processing speed	Symbol search
	Digit Symbol Coding Test
	Trail Making Test A
	Modified Stroop (conditions #1 and #2)
Inhibition	Modified Stroop (condition #3)
Flexibility	Modified Stroop (condition #4)
	Trail Making Test B
Working memory	Letter-Number Sequencing

The Verbal Fluency test (Spring and Benton 1997) consists in saying the largest amount of words possible starting with either the letter “P”, “T”, or “L”. For each letter, 90 seconds are allowed.

In the Wechsler Adult Intelligence Scale-III (WAIS-III) (Wechsler 1981) Similarities subtest, participants are asked to say according to their own judgement, what is similar between two objects or concepts (e.g., a car and a boat). Moreover, the WAIS-III Matrix Reasoning subtest consists in giving visual series of logic that the participants have to complete by selecting the right answer in a sample of five possible answers.

In the WAIS-III Digit Span subtest, participants are only given numbers. In the first part on the test, the participants have to repeat them in the same order as they are given and there is up to 9 digits, and in the second part of the test, they have to recapitulate the digits backwards and there’s up to 8 digits in this case.

The WAIS-III Symbol Search and Digit Symbol Coding subtests both measure psychomotor processing speed. In the Symbol Search subtest, participants have 2 minutes to verify if one of two symbols is present or not in a group of five symbols, while in the Digit Symbol Coding subtest, participants have 2 minutes to match the right symbol to a specific number from a list.

The Trail Making Test Part A of the Halstead-Reitan Neuropsychological Test Battery (Reitan and Wolfson 1985) consists in linking numbers 1 to 25 in ascending order as rapidly as possible and without lifting the pencil. Part B of the Trail Making Test consists in linking numbers in ascending order (1 to 13) and letters in alphabetical order (A to L) by alternating between them. Both tests were timed with a stopwatch.

The modified version of the Stroop includes four conditions (Bohnen et al. 1992). The first condition, i.e., Reading Condition, is to read the words “blue”, “red”, “yellow”, or “green” as fast as possible. The second condition (Denomination) is to name small rectangles of colours that are blue, red or green as fast as possible again. The third condition (Inhibition) is to name as fast as possible, the printed colours of the words “blue”, “red”, “yellow”, or “green”, which all have an incongruent semantic meaning with the actual colour of the word, thus creating interference. Finally, the fourth condition (Flexibility) is to name as fast as possible the printed colours of the words “blue”, “red”, “yellow”, or “green”, which again all have a different semantic meaning than the word itself, except when these words are placed inside a rectangle. Then, the participant has to read the word instead. Consequently, this last condition requires flexibility. All conditions of the Stroop are timed with a stopwatch.

Finally, in the Letter-Number Sequence subtest of the WAIS-III, a series of letters and numbers (up to a total of 8 digits) is given to the participants which are then asked to first reorder the numbers in ascending order and second, to reorder the letters in alphabetical order.

#### PRP training - stimuli and apparatus

The first task (hereafter referred to as task 1) was a visual two-choice reaction time task and participants had to identify whether the stimulus was a digit or letter, which would be chosen from the ensemble “1, 2, 3, 4, A, B, C, or D”, presented in Times New Roman font. The background was white and the characters were black. Viewing distance was of 46 cm and the characters subtended approximately 1.49° vertically by 1.04° horizontally. The second task (Task 2) was an auditory two-choice reaction time task and participants had to identify one of two possible tones presented for a duration of 150 ms: a high tone in pitch (1800 Hz) and a low tone in pitch (400 Hz). The auditory stimuli were presented via headphones with a microphone and a volume control, in case it needed to be adjusted, although it was set to a constant level by default. Stimulus presentation and timing were performed by a PC-compatible computer equipped with a software trigger for detecting speech onset and Chant Speechkit v.4 (Microsoft Speech SDK v.5.1) for recognizing speech.

During the first training phase, each of the 6 training sessions with task Task 2 consisted of 840 experimental trials, for a total of 5,040 trials. The session was broken into fourteen blocks of 60 trials, separated by breaks. During each break, the computer provided feedback on the averaged speed and averaged accuracy of Task 2. Participants were instructed to respond as quickly and accurately as possible.

As for the second training phase, all participants performed three PRP paradigm sessions, pairing the unpracticed Task 1 with the highly practiced Task 2. In this PRP paradigm, the letter or digit (task 1) appeared in the centre of the screen of the screen and the Task 2 tone appeared after a variable SOA (15, 65, 150, 250, 550, or 1,000 ms). Each session consisted of 20 warm-up trials followed by 384 experimental trials (for a total of 1,152 trials). The session was broken into eight blocks of 48 trials, separated by breaks. During each break, the computer provided feedback on the average speed of Task 1 and the accuracy of both Task 1 and Task 2. Participants were instructed to respond as quickly and accurately as possible to each task while emphasizing the speed of Task 1 responses.

After each trial, a message, displayed for 600 ms, informed participants whether they made an erroneous or correct response to the two tasks in the PRP paradigm, or only for Task 2 in the first training phase. Also, if the participant responded within 100 ms of the stimulus onset (either the tone or the character) or failed to respond within 2,500 ms of the stimulus onset (either the tone or the character), a “too early” or a “too slow” message was displayed respectively and the trial was considered as an error. A more detailed description of the procedure of the PRP training is provided in Maquestiaux et al. ( 2008).

### Statistical analysis

To explore individual differences in PRPs, we first calculated PRP values for all participants and create groups according to their individual results. These groups were then compared on their performances on neuropsychological tests. Furthermore, we explored the impact of gender on the different PRP values and neuropsychological tests. For each test, one-way ANOVAs were computed to compare groups on their performances. Finally, to understand which variables contribute the most to explain PRP variance, we performed a backward multiple regression. As reported in Maquestiaux et al. ( 2008), grouped responses (RT2 minus RT1 < 100ms) were eliminated from the analyses, which led to the removal of 4,55% of the trials. To further verify whether grouping occurred, a 6 X 2 mixed design repeated ANOVA with SOAs (15, 65, 150, 250, 550 and 1000 ms) and PRP groups (small and large) as factors using RT1 did not reveal any interaction between the two factors, F(5, 90) = 2,14, p >.05, or any between group effect between the small and large PRP groups, F(1, 18) = 1.61, p >.05. Thus, we feel safe to say that response grouping did not occur or was taken out.

## Abbreviations

ANOVA: Analysis of variance
Hz: Hertz
ms: Milliseconds
PRP: Psychological refractory period
RT1: Response time to task 1
RT2: Response time to task 2
RT: Response times
SOA: Stimulus onset asynchrony
WAIS-III: Wechsler adult intelligence scale-III.
